# Liupao Tea Relieves Constipation by Activating the PI3K/Akt Pathway to Down‐Regulate AQP3 and Restore Gut Microbiota

**DOI:** 10.1002/fsn3.71298

**Published:** 2025-11-28

**Authors:** Wenxin Yu, Jing Xue, Zehua Yang, Xiaoxin Xie, Yi Feng, Yu Zeng

**Affiliations:** ^1^ School of Chinese Materia Medica Guangdong Pharmaceutical University Guangzhou China; ^2^ Department of Pharmacy Zibo Integrated Traditional Chinese and Western Medicine Hospital Zibo China; ^3^ Wuzhou Vocational College Wuzhou Guangxi China; ^4^ The Department of Pharmacokinetics, The Second Affiliated Hospital of Guangzhou University of Chinese Medicine Guangzhou Guangdong China; ^5^ State Key Laboratory of Dampness Syndrome of Chinese Medicine, The Second Affiliated Hospital of Guangzhou University of Chinese Medicine Guangzhou China

**Keywords:** AQP3, constipation, Liupao tea, PI3K/Akt pathway

## Abstract

Due to modern lifestyles and dietary changes, the prevalence of constipation is rising and affecting younger populations. Abnormal intestinal water transport is a major factor contributing to constipation, with aquaporins (AQPs) being crucial for water transfer. Previous studies have indicated that dark tea can effectively relieve constipation. Liupao tea, a type of dark tea from Guangxi, is one such tea, but its mechanisms for alleviating constipation remain to be further investigated. This study will use ultra‐performance liquid chromatography–tandem mass spectrometry (UPLC–MS/MS) combined with network pharmacology to predict the potential targets and pathways through which Liupao tea alleviates constipation. A constipation rat model will be established by simulating multiple factors that induce constipation in modern life. Hematoxylin and eosin (HE) staining will be used to assess pathological changes in the colon, and immunohistochemistry will measure the expression of inflammatory factors in the colon. High‐throughput 16S rRNA sequencing will analyze the effect of Liupao tea on gut microbiota. Real‐time quantitative PCR will quantify AQP3 expression in colon tissues, and Western blotting will verify the regulatory effects of Liupao tea on AQP3 protein and the PI3K/Akt pathway. Network pharmacology predictions suggest that Liupao tea may alleviate constipation by modulating the PI3K/Akt pathway. Experimental results show that Liupao tea significantly increases fecal water content in constipated rats and reduces the levels of TNF‐α, IL‐6, and IL‐1β in the colon and stomach tissues. Western blot analysis confirms that Liupao tea significantly enhances Akt phosphorylation and decreases AQP3 expression. Additionally, Liupao tea increases the abundance of gut microbiota in constipated rats. Correlation analysis suggests that Liupao tea may alleviate constipation by improving amino acid metabolism through modulation of the gut microbiota. This study suggests that Liupao tea may alleviate constipation by activating the PI3K/Akt pathway, inhibiting AQP3 expression, and regulating gut microbiota to improve amino acid metabolism.

## Graphical abstract

Liupao tea, a Guangxi dark tea, alleviates constipation in rats by increasing fecal water content, reducing pro‐inflammatory cytokines (TNF‐α, IL‐6, IL‐1β) in the colon and stomach tissues. Western blot analysis confirms that Liupao tea activates the PI3K/Akt pathway while suppressing AQP3 expression. Correlation analysis suggests that Liupao tea may alleviate constipation by improving amino acid metabolism through modulation of the gut microbiota.

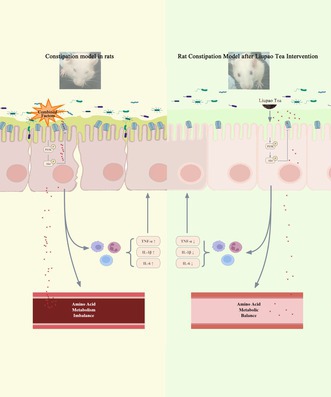

**Abbreviations**
Akt: Protein kinase B
AQP: Aquaporins
CAS: Chemical Abstracts Service
GO: Gene ontology
KEGG: Kyoto Encyclopedia of Genes and Genomes
LT: Liupao tea
LTWE: Liupao tea water extract
PI3K: Phosphatidylinositol 3‐kinase
PPI: Protein–protein interaction
TCM: Traditional Chinese Medicine

## 1 Introduction

Constipation is a common digestive system disorder caused by multiple factors, primarily characterized by reduced frequency of bowel movements, hard stools, straining during defecation, and a feeling of incomplete evacuation (Gao et al. 2021). Constipation is influenced by factors such as diet, stress, anxiety, and environmental conditions (Punukollu et al. 2024 and Lu et al. 2025), though its underlying mechanisms are not fully understood. Modern research has indicated that constipation can often result from intestinal oxidative stress and immune imbalance, disruption of gut microbiota homeostasis, abnormal intestinal water transport, and gastrointestinal motility disorders (Drossman 2016; Evans and Kalman 2024). Epidemiological studies show that constipation is prevalent worldwide and is becoming increasingly common among younger populations due to the accelerating pace of life and increasing stress (Bangran et al. 2023; Xin et al. 2023).

In recent years, studies suggest that Liupao tea (LT) might be a safe and effective food for addressing constipation. Liupao tea is a representative type of dark tea known for its anti‐inflammatory, antioxidant, gut microbiota‐regulating, and gastrointestinal function‐improving properties. LT extracts have been shown to reduce serum levels of inflammatory cytokines TNF‐α, IFN‐γ, and IL‐1β in obese mice, while increasing serum levels of anti‐inflammatory cytokines IL‐10 and IL‐4 (Ya et al. 2020). Liupao tea polysaccharides (Siqi et al. 2022) have strong antioxidant properties, with compounds like tannic acid, hypoxanthine, and theobromine showing significant positive correlations with antioxidant activity, and exhibiting better antioxidant activity compared to green tea (Shuoyuan et al. 2022). Furthermore, research by Waijiao Tang and colleagues has shown that LT water extracts can increase the richness and diversity of the microbiota in mice fed a high‐fat diet (Waijiao et al. 2022). Traditionally, dark tea has been used to improve gastrointestinal motility, and studies by Zhi‐ping Gong and others have indicated that LT extracts can promote gastric emptying and accelerate small intestinal transit in normal rats (Gong et al. 2020). Thus, LT may emerge as a food with medicinal properties that can effectively relieve constipation (Wenliang et al. 2017).

Aquaporins (AQPs) are a family of membrane proteins with high water permeability, playing a crucial role in the reabsorption of water in the gastrointestinal tract. Various AQP family members regulate intestinal water metabolism, thereby controlling fecal water content (M. Liu et al. 2024). AQP3, in particular, is expressed primarily in the colonic mucosal epithelial cells and plays an important role in colonic water transport. Upregulation of AQP3 expression may disrupt the colonic water transport system, potentially leading to constipation (Cai et al. 2024; Ikarashi et al. 2016). Therefore, investigating the regulatory effects of LT on AQPs can help elucidate its potential mechanisms for improving constipation.

In this study, we used a database of LT water extracts established by our research group to analyze relevant targets and compare them with constipation‐related targets, thereby identifying the pathways involved in LT's effect on constipation. Western blot experiments revealed that LT downregulates AQP3 protein expression and upregulates the expression levels of p‐PI3K and p‐Akt. Based on these results, we hypothesize that LT may alleviate constipation symptoms by inhibiting inflammatory factors, regulating gut microbiota, activating the PI3K/Akt signaling pathway, and downregulating AQP3 expression.

## 2 Materials and Methods

### 2.1 Materials

Liupao tea (LT) was obtained from the Wuzhou Tea Factory in Guangxi Zhuang Autonomous Region. It was soaked in ultrapure water for 1 h, followed by heat reflux extraction for 1.5 h (material‐to‐liquid ratio 1:10). The mixture was filtered, and the residue was extracted again using the same method. The two filtrates were combined, concentrated, and freeze‐dried to obtain LT water extract powder (Danshui et al. 2023). Chromatographic‐grade methanol was purchased from Merck, distilled water from Watsons, and lard from Zhejiang Jinen Food Technology Co. Ltd. Primary antibodies for interleukin‐6 (IL‐6), tumor necrosis factor‐alpha (TNF‐α), and interleukin‐1 beta (IL‐1β) were provided by Wuhan Sevier Biotechnology Co. Ltd.

### 2.2 Instruments

#### 2.2.1 Liquid Chromatography Conditions

The liquid chromatography‐mass spectrometry system used Waters Acquity UPLC HSS T3 columns (50 × 2.1 mm, 1.8 μm, Thermo Fisher Scientific), coupled with a Thermo Fisher UPLC system for sample analysis. The mobile phases included phase A (0.1% formic acid in water) and phase D (0.1% formic acid in acetonitrile). The gradient elution was as follows: 0–3 min, 5% D; 3–42 min, 5%–95% D; 42–47 min, 95% D; 47–47.1 min, 95%–5% D; 47.1–50 min, 5% D. The injection volume was 1 μL, and the flow rate was 0.3 mL/min.

#### 2.2.2 Mass Spectrometry Conditions

Mass spectrometry data were acquired using a QE mass spectrometer equipped with an ESI source. The ESI source parameters were set as follows: spray voltage at 3500 V, sheath gas pressure at 35 psi, auxiliary gas pressure at 10 psi, ion transfer tube temperature at 320°C, and auxiliary gas temperature at 350°C. The drying gas temperature was set at 300°C with a flow rate of 8 mL/min, nebulizer gas pressure at 172 kPa (25 psi), fragmentor voltage at 180 V, and the scanning range from m/z 200 to 1000.

### 2.3 Identification of LT Constituents

#### 2.3.1 Preparation of LT Water Extract Samples

Precisely weigh 1.0 mg of LT water extract powder and dissolve it in 50% methanol to prepare a 0.1 mg/mL solution. The solution was filtered through a 0.22 μm organic filter and then analyzed by injection.

#### 2.3.2 Data Analysis

The results were analyzed using Tracefinder 3.3 software.

### 2.4 Network Pharmacology Analysis

Based on previous liquid chromatography‐mass spectrometry results for LT water extracts (Wu et al. 2023), and using the current LC–MS results as supplementary data, the TCMSP database was utilized to collect the chemical constituents of LT with oral bioavailability (OB) ≥ 30% as the screening criterion, identifying them as LT's active components. Targets for these active components were predicted using TCMSP and SymMap databases. Gene names were converted from protein names using the Uniprot database to collect the action targets of LT active components.

Disease‐related targets for constipation were retrieved using the keyword “Constipation” from OMIM, NCBI, DrugBank Database, GeneCards, and SymMap databases. The intersection of constipation‐related targets and active component targets was determined to identify potential targets for LT in improving constipation.

Protein–protein interaction analysis of potential targets was performed using the STRING database, and key targets and components were selected using Cytoscape 3.7.1 software. Enrichment analysis of potential targets was conducted using the DAVID database and Metascape online platform, and the results were visualized to further elucidate the mechanism of LT in improving constipation (URLs mentioned above are listed in Table 1).

**TABLE 1:** Database.

Name	URL
Traditional Chinese Medicine Systems Pharmacology Database and Analysis Platform (TCMSP)	https://old.tcmsp‐e.com/tcmsp.php
SymMap	http://www.symmap.org/
Uniprot	https://www.uniprot.org/
OMIM	http://www.omim.org/
NCBI	https://www.ncbi.nlm.nih.gov/
Drugbank Database	https://go.drugbank.com/
GeneCards	https://www.genecards.org/
STRING	https://cn.string‐db.org/
The Database for Annotation, Visualization and Integrated Discovery (DAVID)	https://david.ncifcrf.gov/
Metascape	http://metascape.org/gp/

### 2.5 Animal Experiments

This study was approved by the Animal Experiment Center of Guangdong Pharmaceutical University (SYXK (Yue) 2017–0125) and endorsed by the Experimental Animal Management and Use Committee of Guangdong Pharmaceutical University, Guangzhou, China (SYXK (Yue) 2018–0002). After a 7‐day acclimatization period, 32 specific pathogen‐free (SPF) SD male rats (180 ± 20 g) were randomly divided into 4 groups (*n* = 8): Control Group (C group), Model Group (M group), Low‐Dose LT Water Extract Group (LD group, 0.063 g tea/100 g body weight), and High‐Dose LT Water Extract Group (HD group, 0.126 g tea/100 g body weight).

The Control Group was housed in an SPF environment (temperature 20°C–25°C; humidity 50%–60%) and provided standard food and normal drinking water. Rats in the Model Group and LT Low‐ and High‐Dose Groups were exposed daily for 12 h in a custom‐made modeling chamber (temperature: 32°C–35°C, humidity 80%–90%) and were fed standard food with free access to 15% honey water. They were fasted for 1 day, with each rat receiving 3 mL of lard via gavage in the morning and subjected to forced exercise in the afternoon until fatigue (unable to maintain the original exercise intensity, running 1/3 of the track more than 3 times). The High‐Dose and Low‐Dose LT Groups received lard via gavage in the morning and LT extract according to dose in the afternoon. The intervention lasted for 5 weeks.

### 2.6 General Observations of Rats

During the experiment, general observations were made and recorded for each group of SD rats, including changes in body weight, coat condition, mental state, and fecal morphology.

### 2.7 Measurement of Fecal Water Content

On the 35th day of modeling (week 4), fecal water content was measured for each group. Feces were collected by gently massaging the rat's abdomen in a clockwise direction and using pre‐dried weighing bottles (weight recorded as M1). After weighing, the sample was recorded as M2. The feces were then dried to a constant weight in a 60°C forced‐air oven and weighed again (M3). The fecal water content was calculated using the formula:

$$\text{Fecal Water Content}\;\left(\%\right)=\left[\left(\text{M2}–\text{M1}\right)–\text{M3}\right]/\left(\text{M2}–\text{M1}\right)$$

### 2.8 Intestinal Propulsion Rate

After a 24‐h fasting period but with free access to water, all SD rats were euthanized. One hour before euthanasia, a semi‐solid paste (composed of sodium carboxymethyl cellulose, skim milk powder, glucose, starch, and a small amount of carbon powder) was administered by gavage at a dose of 1 mL/100 g body weight. Thirty minutes later, rats were anesthetized with an intraperitoneal injection of 20% urethane (1 mg/g). The small intestine from the pylorus to the cecum was excised, and the mesentery was carefully removed. The intestine was laid out on white paper, and the lengths from the pylorus to the cecum (L1) and from the pylorus to the front edge of the semi‐solid paste (L2) were measured.

$$\text{Intestinal Propulsion Rate}\;\left(\%\right)=\left(L2/\text{L1}\right)\times 100\%$$

### 2.9 Hematoxylin–Eosin and Immunohistochemical Staining

After dissection, colon and stomach tissues were subjected to routine HE staining to observe pathological changes. The pathological damage index of gastric mucosa in rats was calculated by Mascuda standard, and the cumulative damage score of each paraffin section was less than 15 points. According to Ding et al.'s literature (Ding and Wen 2018), colon histological score was made. Immunohistochemical methods were used to localize, quantify, and analyze the expression of TNF‐α (Rabbit, 1:500, Servicebio, GB11188), IL‐6 (Rabbit, 1:1000, Servicebio, GB11117), IL‐1β (Rabbit, 1:1000, Servicebio, GB11113), and AQP3 (Rabbit, 1:1000, Servicebio, GB11395) proteins in colon and stomach tissues. The protein expression locations and levels were observed under a microscope after mounting.

### 2.10 Real‐Time Quantitative PCR

Real‐time quantitative PCR was used to detect AQP3 gene expression levels in colon tissues. RNA was extracted using the Trizol method, and RNA concentration and A260/A280 ratios were measured with a spectrophotometer. RNA concentrations were adjusted to 1 μg/μL, and reverse transcription and real‐time PCR were performed according to the manufacturer's instructions. GAPDH (Sangon Biotech) was used as an internal control, and data were processed using the $2^{-△△C_{t}}$ method. Primer sequences are detailed in Table 2.

**TABLE 2:** Primer sequences.

Gene	Forward primer (5′–3′)	Revers primer (5′–3′)
AQP3	CTGTGGTTCCGTGGCTCAAGTG	GATGGCAAGGGTGACAGCGAAG
GAPDH	ATTGGGCGCCTGGTCAC	CCAGAGGGGCCATCCAC

### 2.11 Western Blot Analysis of AQP3, PI3K, Akt, p‐PI3K, and p‐Akt Proteins

Colon tissues from each group were homogenized to extract total proteins, and protein concentrations were measured according to the instructions provided with the protein assay kit. The protein concentrations were adjusted to a uniform level across all groups. Following this, 5× SDS sample buffer was added, and the samples were boiled at 100°C for 10 min to denature the proteins. Equal amounts of protein were separated on 10%–12% SDS‐PAGE gel (Beyotime, P0012A). After membrane transfer, the membrane was sealed with 5% skim milk at room temperature for 1 h, and then incubated with primary antibody at 4°C overnight. Western blotting was performed to detect the expression of AQP3 (Rabbit, 1:1000, Beyotime, AF6222), PI3K (Rabbit, 1:1000, Affinity, AF6242), Akt (Rabbit, 1:1000, Cell Signaling, 9272), p‐PI3K (Rabbit, 1:1000, Affinity, AF3242), and p‐Akt (Rabbit, 1:1000, Cell Signaling, 9271), β‐Actin (Rabbit, 1:2000, Beyotime, AF5003). Subsequently, the membrane was incubated with the second antibody for 1 h, and the resulting protein‐imprinted image was imaged and analyzed using Super Sensitive ECL Luminescence Reagent (Meilunbio, MA0186). Protein expression was quantified using ImageJ software.

### 2.12 Gut Microbiota Analysis Based on 16S rRNA High‐Throughput Sequencing

After dissection, colonic and cecal content samples were collected and stored at −80°C for later use. Genomic DNA was extracted from the intestinal contents using the QIAamp Fast DNA Stool Mini Kit, and its integrity and purity were assessed. PCR amplification of the 16S rRNA gene V3–V4 regions was carried out using specific primers. The amplification products were purified using magnetic beads, quantified, and then pooled to construct the sequencing library. The qualified libraries were sequenced on an Illumina HiSeq 2500 platform using paired‐end sequencing methods. The sequencing was conducted by Shanghai Meiji Biomedical Technology Co. Ltd. (Table 3).

**TABLE 3:** Specific primer sequences.

Primer name	Primer sequence
338F	ACTCCTACGGGAGGCAGCAG
806R	GGACTACHVGGGTWTCTAAT

### 2.13 Data Analysis

Unless otherwise specified, all results are presented as mean ± standard error of the mean (SEM). Data were analyzed using GraphPad Prism 9.4 software, with results expressed as mean ± SEM. One‐way ANOVA was used for statistical analysis, with multiple comparisons conducted using the Tukey method. A *p*‐value < 0.05 was considered to indicate a statistically significant difference between groups.

## 3 Results

### 3.1 Major Components of LT

**FIGURE 1:**
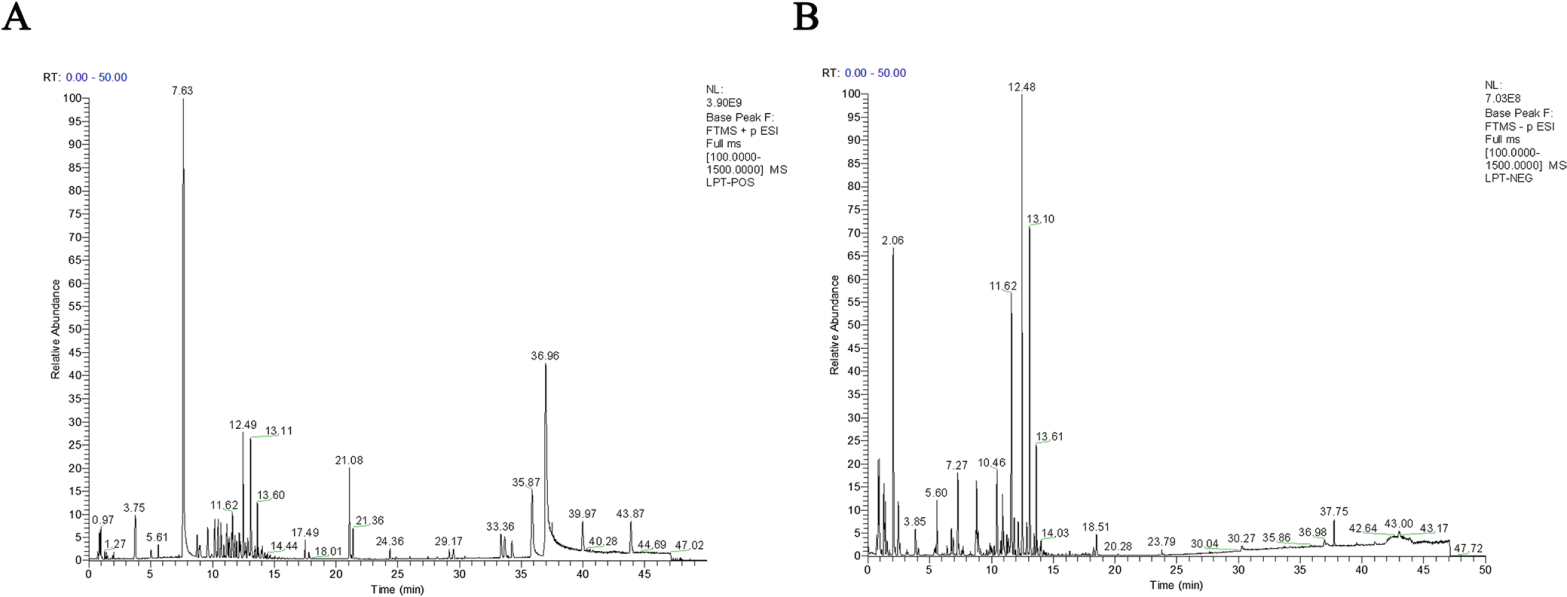
Total ion chromatogram (TIC) of LTWE. (A) Positive mode. (B) Negative mode.

Identification of LT components was performed by comparing liquid chromatography‐mass spectrometry (LC–MS) results with database and literature references. Figure 1 shows the total ion chromatogram of positive and negative ions of LTWE. Components with a Fragment Ion (FI) status of PASS and an Oral Bioavailability (OB) value > 30% were selected (Table 4). These results, combined with previous research on the same batch of LT conducted by our research team, identified the active components of LT.

**TABLE 4:** Main components of LT water extract (OB ≥ 30%).

FI	Compound name	Formula	Adduct	m/z (Apex)	RT (Measured)	OB (%)
Pass	6‐Gingerol	C17H26O4	M‐H	293.18	22.47	35.64
Pass	5‐Hydroxymethylfurfural	C6H6O3	M + H	127.04	1.76	45.07
Pass	5‐Hydroxytryptophan	C11H12N2O3	M + H	221.09	6.27	63.93
Pass	Alpha‐Linolenic acid	C18H30O2	M + H	279.23	18.26	45.01
Pass	Cianidanol	C15H14O6	M + H	291.09	7.28	54.83
			M‐H	289.07	7.27	
Pass	Dibutyl Phthalate	C16H22O4	M + H	279.16	31.54	64.54
Pass	Eriodictyol	C15H12O6	M + H	289.07	7.29	71.79
Pass	Fisetin	C15H10O6	M + H	287.05	11.83	52.6
Pass	Herbacetin	C15H10O7	M + H	303.05	10.89	36.07
Pass	Isovitexin	C21H20O10	M + H	433.11	11.03	31.29
			M‐H	431.1	11.02	
Pass	L‐Isoleucine	C6H13NO2	M + H	132.1	1.66	59.05
Pass	L‐Leucine	C6H13NO2	M + H	132.1	1.66	72.92
Pass	L‐Phenylalanine	C9H11NO2	M + H	166.09	2.69	41.62
Pass	Luteolin	C15H10O6	M + H	287.05	11.83	36.16
Pass	L‐Valine	C5H11NO2	M + H	118.09	0.87	53.33
Pass	Morin	C15H10O7	M + H	303.05	10.89	46.23
Pass	Protocatechualdehyde	C7H6O3	M + H	139.04	7.28	38.35
Pass	Quercetin	C15H10O7	M + H	303.05	10.89	46.43

### 3.2 Prediction of LT Targets and Pathways for Relieving Constipation

**FIGURE 2:**
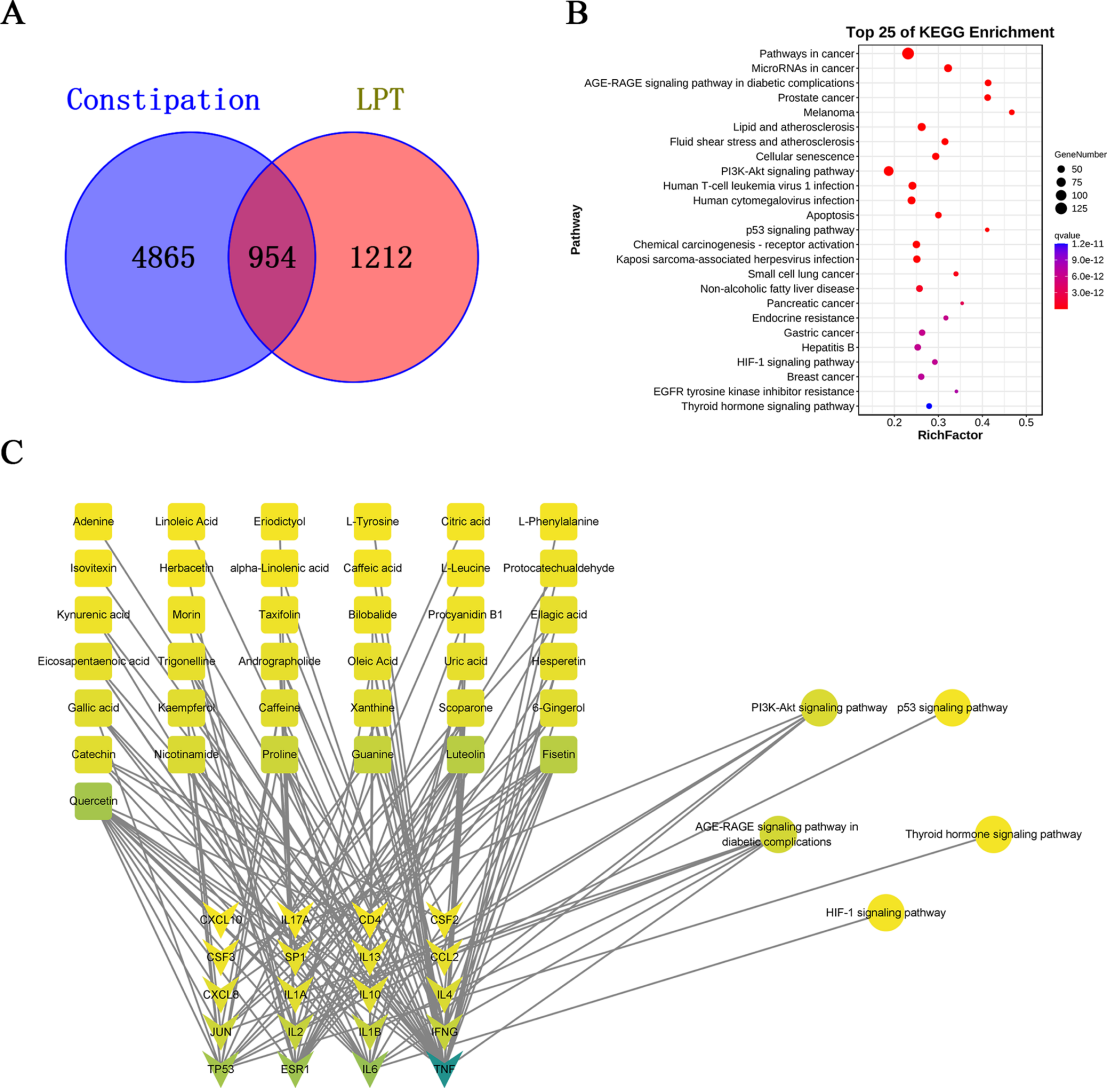
Network pharmacological results of LT in improving constipation. (A) Venn diagram of constipation target and LT target. (B) KEGG enrichment analysis diagram of the potential target of LT in improving constipation. (C) “Composition‐Target‐Pathway” Diagram of LT in Improving Constipation.

A total of 2166 targets were identified for LT active components, and 5819 targets were associated with constipation. The intersection of these targets yielded 954 potential targets for LT's effect on constipation (Figure 2A).

Protein–protein interaction analysis of these potential targets was performed, and the data were imported into Cytoscape software for further analysis. The CytoHubba plugin was used for visualization, and the top 20 targets based on MCC (Maximum Clique Centrality) values were selected as key targets (Zhang et al. 2024). A component‐key target‐pathway network diagram was constructed, and KEGG enrichment analysis was conducted for the potential targets of LT in improving constipation. According to KEGG analysis, there are 152 pathways with *q* < 0.01. Select the top 25 entries to draw a bubble chart. The bubble size represents the number of genes enriched in the pathway, and the color difference represents the enrichment degree, as shown in Figure 2B. The main signal paths involved in the figure include age‐range signaling pathway in diabetic complications, lipid and atherosclerosis, PI3K‐Akt signaling pathway, etc. The results indicated that the potential targets were involved in multiple related pathways, suggesting that LT acts on constipation through multiple components, targets, and pathways (Figure 2C).

### 3.3 LT Improves Constipation Symptoms in Rats

Rats in the Control Group displayed healthy fur with a shiny appearance, were responsive and active, and had normal food and water intake, with a steady increase in body weight. In contrast, rats in the Model Group showed yellowed, less glossy fur, reduced food intake, and significant weight loss (*p* < 0.05). These rats were lethargic, preferred to lie down, and exhibited signs of huddling and arching their backs. When touched, they appeared tense and irritable, and their feces were smaller and drier.

After intervention with LT, both the Low‐Dose and High‐Dose groups showed improvements in general condition and activity levels compared to the Model Group.

**FIGURE 3:**
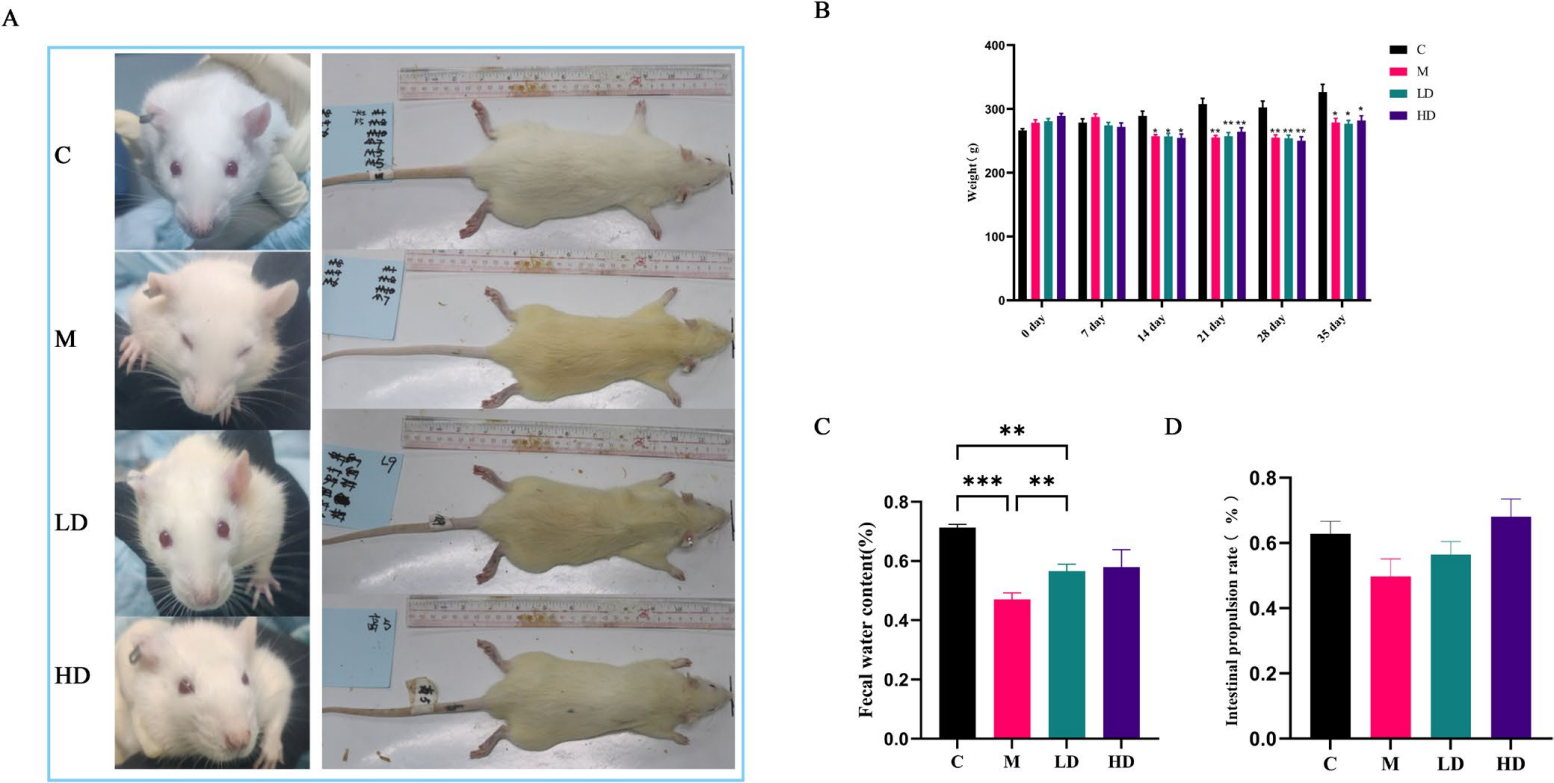
Effects of LT on (A) Fur, (B) Weight, (C) Fecal water content, (D) Intestinal propulsion rate in constipation rats. Data are expressed as the mean ± SEM, *n* = 6. *p < 0.05, **p < 0.01, ***p < 0.001.

By the fourth week of modeling (day 35), rats in the Model Group exhibited notably yellowed fur, significant weight loss, and lethargy (Figure 3A,B). As shown in the results, the fecal water content in the Model Group was significantly lower compared to the Control Group (*p* < 0.001). Both LT treatment groups significantly increased fecal water content in constipated rats (*p* < 0.01) (Figure 3C). The intestinal propulsion rate results indicate that while the Model Group had a reduced intestinal propulsion rate compared to the Control Group (*p* > 0.05), the intestinal propulsion rates in the LT treatment groups were higher compared to the Model Group (*p* > 0.05), though this difference was not statistically significant (Figure 3D).

### 3.4 LT Alleviates Pathological Symptoms in Constipated Rats

**FIGURE 4:**
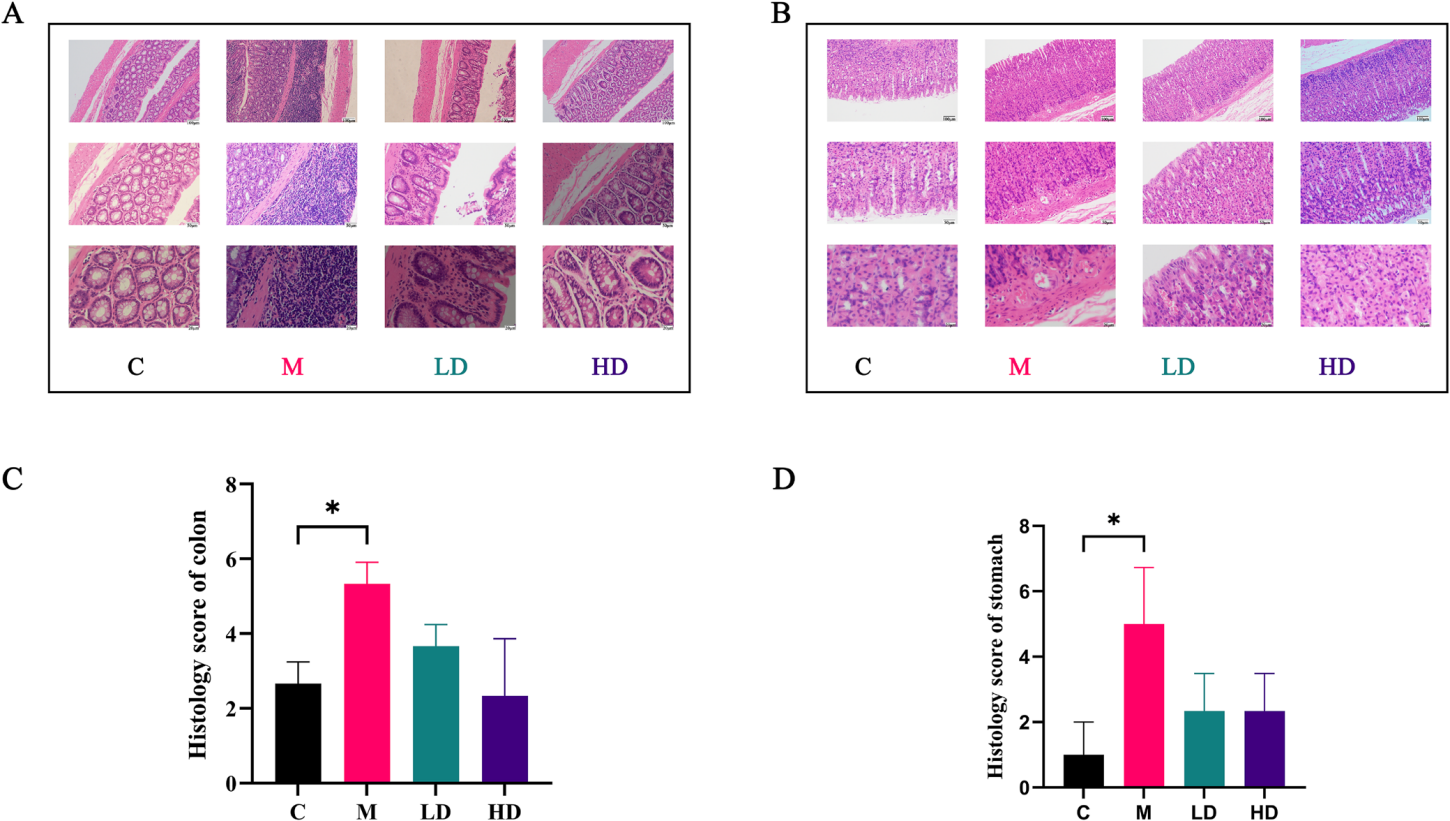
Morphological damages. (A) Colonic tissues. (B) Gastric tissues. (C) Histology score of colon. (D) Histology score of stomach (*n* = 3, 100×, 200×, 400×).

HE staining results showed that compared to the Control Group, the colon of the Model Group rats exhibited a slightly smoother mucosal surface, with atrophy of intestinal glands, disorganized and ruptured goblet cells. Lymphocyte aggregation was observed in the submucosa, structural disorganization was evident, and the thickness of the muscle layer was significantly reduced. However, in the LT Low‐Dose and High‐Dose groups, these pathological features were alleviated. The cell arrangement disorder was improved, the number of lymphocytes in the submucosa was reduced, and the muscle layer thickness was increased (Figure 4A,C).

Regarding gastric tissue, compared to the Control Group, the gastric mucosal epithelial cells in the Model Group rats showed mild desquamation, and there was inflammatory cell infiltration between the lamina propria and the muscularis mucosa. In contrast, the LT Low‐Dose Group exhibited reduced desquamation of gastric mucosal epithelial cells, though inflammatory cell infiltration between the lamina propria and the muscularis mucosa was still present. In the High‐Dose Group, while there was still some epithelial cell desquamation, significant inflammatory cell infiltration between the lamina propria and the muscularis mucosa was not observed (Figure 4B,D).

### 3.5 LT Reduces Inflammatory Factor Levels in the Colon and Gastric Tissue of Constipated Rats

TNF‐α, IL‐1β, and IL‐6 are secretory inflammatory factors. The expression levels of these factors are indicated by varying degrees of brown‐yellow or brown‐brown coloration at the cell membranes or inflammatory sites.

**FIGURE 5:**
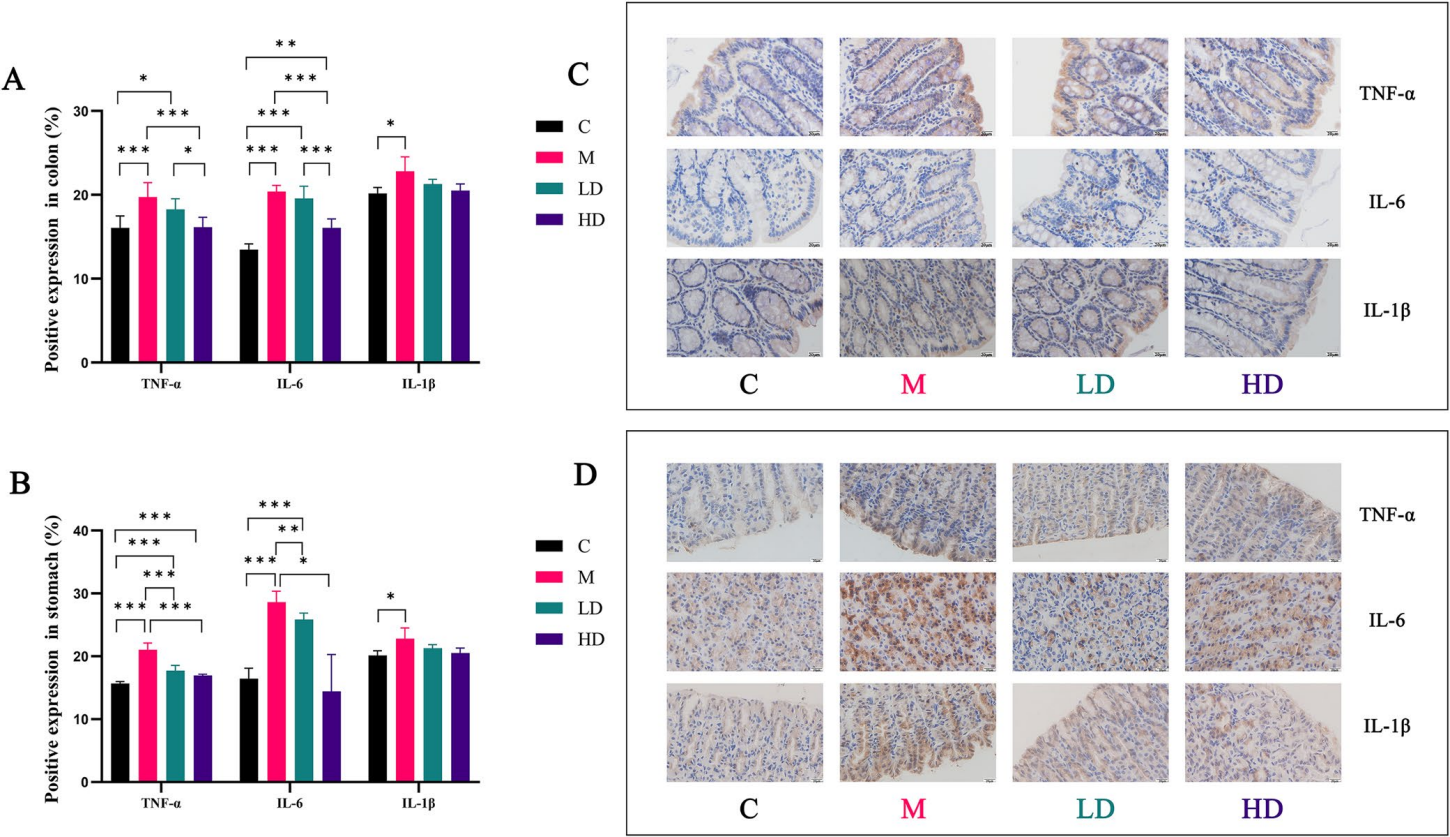
Pro‐inflammatory cytokines expression in (A, C) colonic tissues and (B, D) gastric tissues. Data are expressed as the mean ± SEM, *n* = 3, 400×. *p < 0.05, **p < 0.01, ***p < 0.001, ****p < 0.0001.

Optical microscopy observations revealed that the Model Group exhibited significantly stronger positive expression of inflammatory factors compared to the other three groups, with the Control Group showing the weakest expression. Semi‐quantitative analysis of the positive expression corroborated the trends observed under the optical microscope. Compared to the Control Group, the Model Group had a significant increase in the expression levels of TNF‐α, IL‐1β, and IL‐6 in both the colon and gastric tissues (*p* < 0.05). In contrast, LT treatment groups showed reduced positive expression of these inflammatory factors compared to the Model Group. Notably, the High‐Dose Liupao Tea group significantly downregulated the expression of TNF‐α and IL‐6 (*p* < 0.05) (Figure 5).

### 3.6 LT Regulates AQP3 Expression in the Colon Tissue of Constipated Rats

**FIGURE 6:**
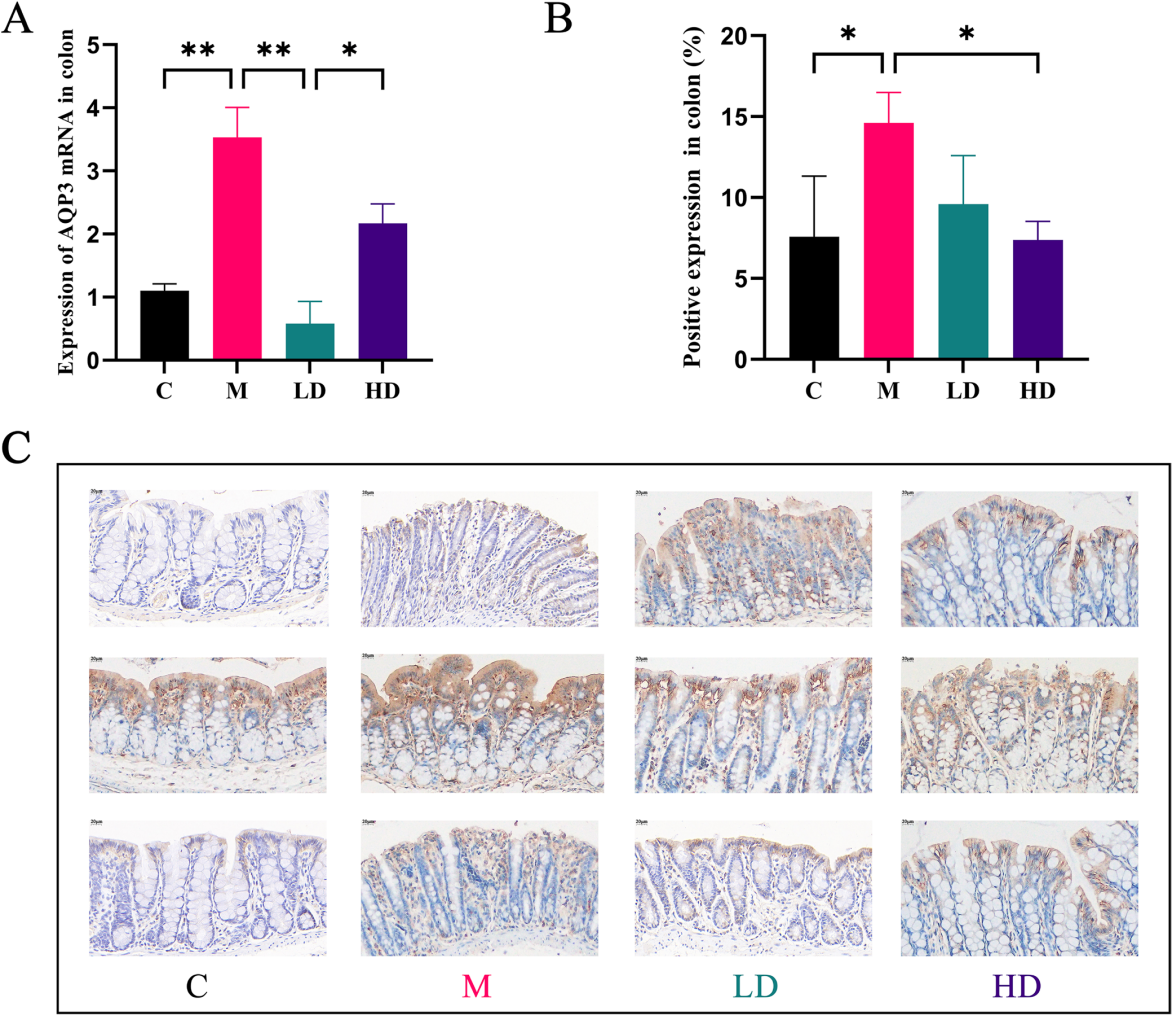
AQP3 expression in colonic tissue. (A) Expression of AQP3 mRNA in colonic tissue. (B) AQP3 expression level in colonic tissue. (C) IHC staining of AQP3 in colonic tissue. Data are expressed as the mean ± SEM, *n* = 3, 400×. *p < 0.05, **p < 0.01.

**FIGURE 7:**
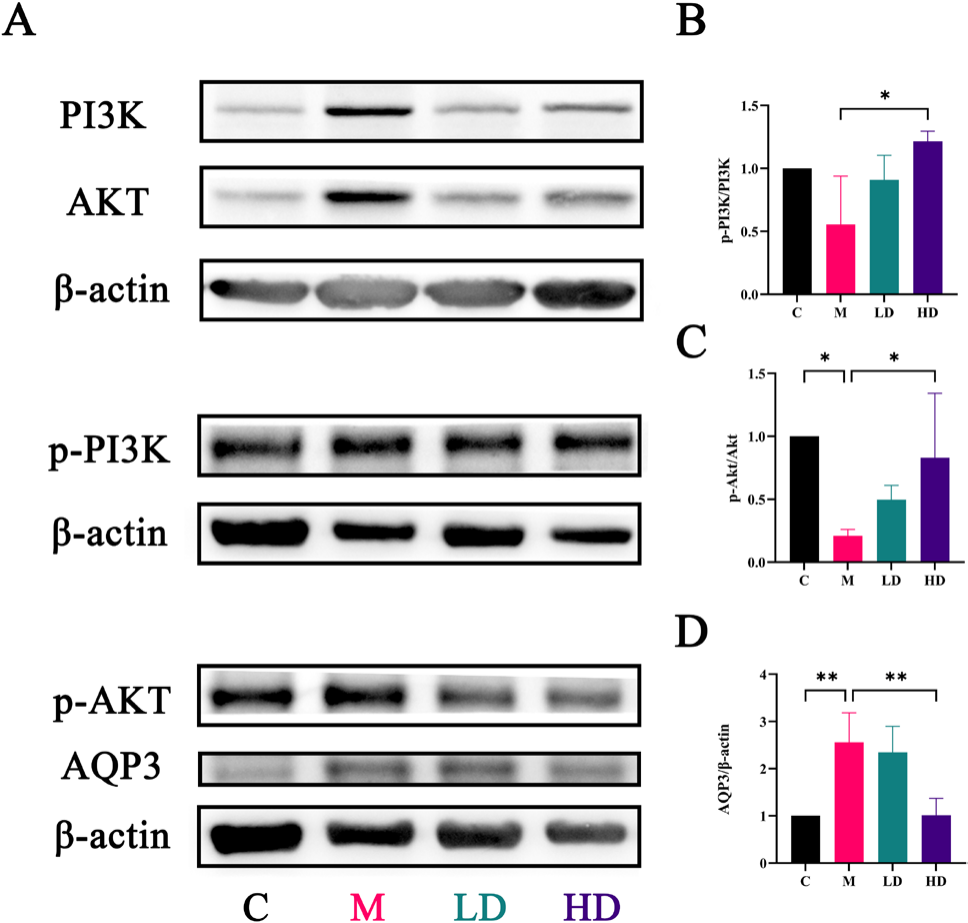
The protein expression levels of PI3K, Akt, p‐PI3K, p‐Akt, AQP3 in the colon tissue. Data are expressed as the mean ± SEM, *n* = 3. *p < 0.05, **p < 0.01.

In the colon tissue, the Model Group rats exhibited significantly higher levels of AQP3 gene expression and corresponding immunohistochemical protein levels compared to the Control Group (*p* < 0.01, *p* < 0.05). This suggests that the overexpression of AQP3 in the constipation model may be associated with the development of constipation. However, after LT intervention, the AQP3 gene expression was significantly downregulated in the Low‐Dose LT Group (*p* < 0.01), and the AQP3 protein levels were significantly reduced in the High‐Dose LT Group (*p* < 0.05). These findings indicate that LT can effectively modulate the overexpression of AQP3, potentially alleviating constipation symptoms through this mechanism. This result suggests that LT may improve the balance of colonic water reabsorption by regulating AQP3 expression, thereby reducing constipation (Figures 6 and 7).

### 3.7 LT May Regulate Aquaporin Expression Through the PI3K‐Akt Signaling Pathway

Western blot analysis was employed to investigate the effects of LT treatment on the expression of p‐PI3K, p‐Akt, and AQP3. As shown in Figure 7, the results indicated that the levels of p‐PI3K and p‐Akt were significantly lower in the colon tissues of the Model Group rats compared to the Control Group (*p* > 0.05 and *p* < 0.05, respectively), whereas the expression of AQP3 was significantly higher (*p* < 0.01). This is consistent with the AQP3 mRNA expression results and immunohistochemical analysis, suggesting that the overexpression of AQP3 in the constipation model is closely associated with the development of constipation.

Following high‐dose LT intervention, the expression levels of p‐PI3K and p‐Akt in the colon tissue were significantly increased (*p* < 0.05), while AQP3 expression was significantly reduced (*p* < 0.01). These data indicate that LT can activate the PI3K‐Akt signaling pathway and alleviate constipation induced by multiple factors by downregulating AQP3 expression in the colon. This supports the mechanism by which LT mitigates constipation symptoms through modulation of the water transport system and improvement of AQP3 overexpression.

### 3.8 LT Improves Gut Microbiota Structure in Constipated Rats

**FIGURE 8:**
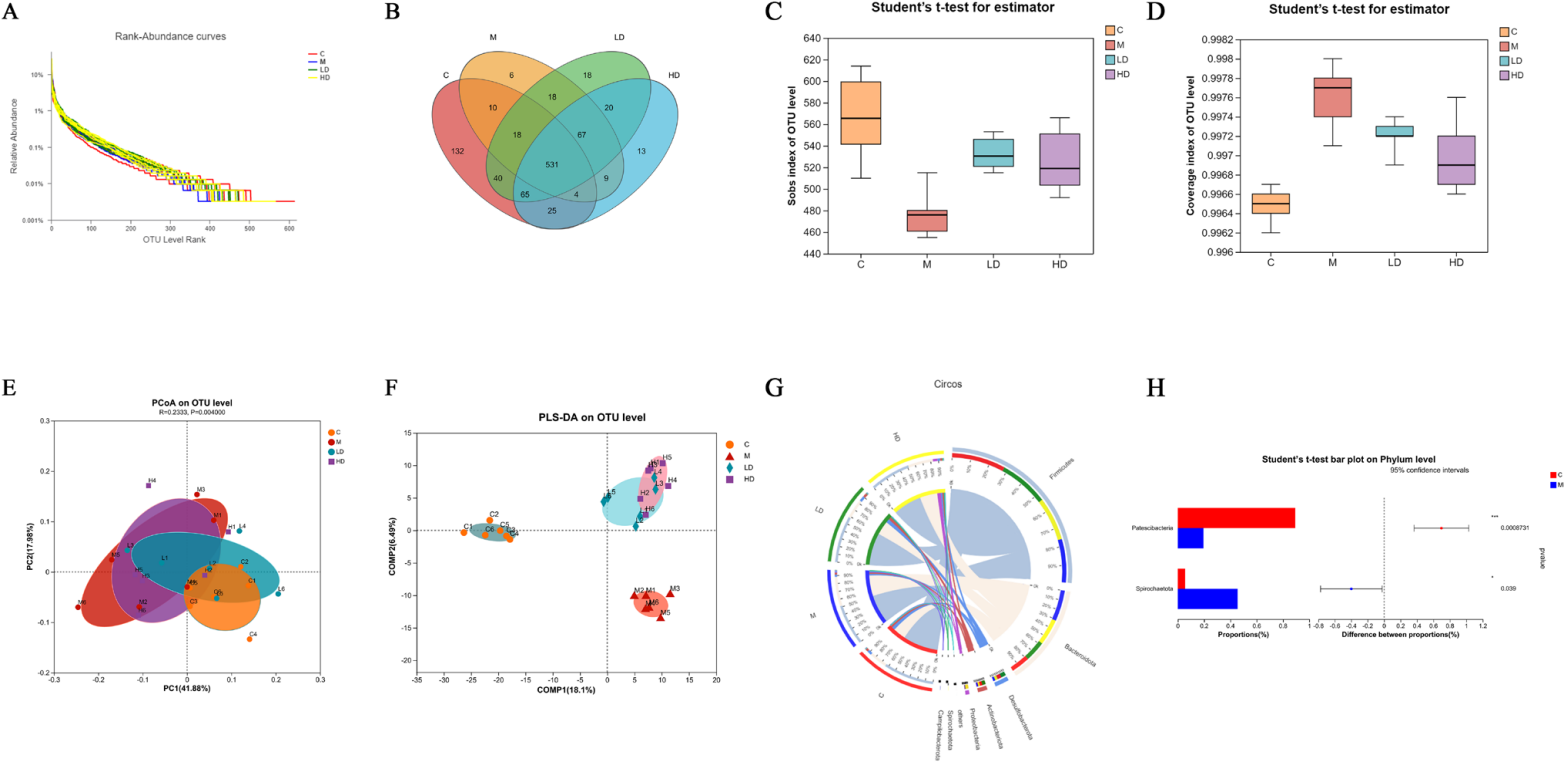
Comparison of gut microbial diversity and composition between groups. (A) Rank‐Abundance curves. (B) Venn diagram of OTUs. (C) Sobs index of OTU level. (D) Coverage index of OTU level. (E) PCoA on OTU level. (F) PLS‐DA on OTU level. (G) Visualization diagram. (H) Phylum with significant difference between group C and group M. Data are expressed as the mean ± SEM, *n* = 6. *p < 0.05, **p < 0.01, ***p < 0.001.

Analysis of the gut microbiota was performed using the Meiji Biotech cloud platform, as shown in Figure 8. Panels A–D indicate that the Model Group had the lowest OTU (Operational Taxonomic Unit) count among the four groups. Compared to the Control Group, the Sob index in the Model Group was significantly reduced (*p* < 0.001), indicating a marked decrease in gut microbiota diversity and abundance in the constipation model rats. After LT intervention, the OTU count increased, and the Sob index was significantly elevated in both the low‐dose and high‐dose LT groups (*p* < 0.01 and *p* < 0.001, respectively). These results suggest that LT effectively restores the disrupted gut microbiota structure under pathological constipation conditions. Figures F and G show the clustering of gut microbiota, with a distinct separation between the Control and Model Groups. The microbiota distribution in the LT low‐dose and high‐dose groups was closer to that of the Control Group. These findings further support the restorative effect of LT on the gut microbiota, suggesting that LT may alleviate constipation by improving the gut microbiota structure.

In terms of species composition, the Firmicutes phylum content in the Model Group decreased (*p* > 0.05), while the Bacteroidetes phylum content increased (*p* > 0.05), resulting in a decreased Firmicutes–Bacteroidetes ratio. LT intervention effectively reversed this trend, restoring the balance between Firmicutes and Bacteroidetes. At the genus level, comparison between the Control and Model Groups, as well as among the four groups, revealed nine genera with significant differences. These differential genera showed consistent patterns across the groups and may serve as potential microbiota markers for LT's effect on constipation. Specifically, compared to the Control Group, the Model Group rats exhibited a significant increase in the genera Bacteroides, Blautia, and Alistipes (*p* < 0.01), while the relative abundance of Romboutsia, Christensenellaceae_R‐7_group, norank_f__norank_o__Clostridia_UCG‐014, and three other genera significantly decreased (*p* < 0.05). Further functional annotation analysis using PICRUSt2 indicated that metabolic functions were the primary biological functions enriched in the gut microbiota. These findings suggest that LT intervention may improve constipation by modulating both the species composition and metabolic functions of the gut microbiota.

**FIGURE 9:**
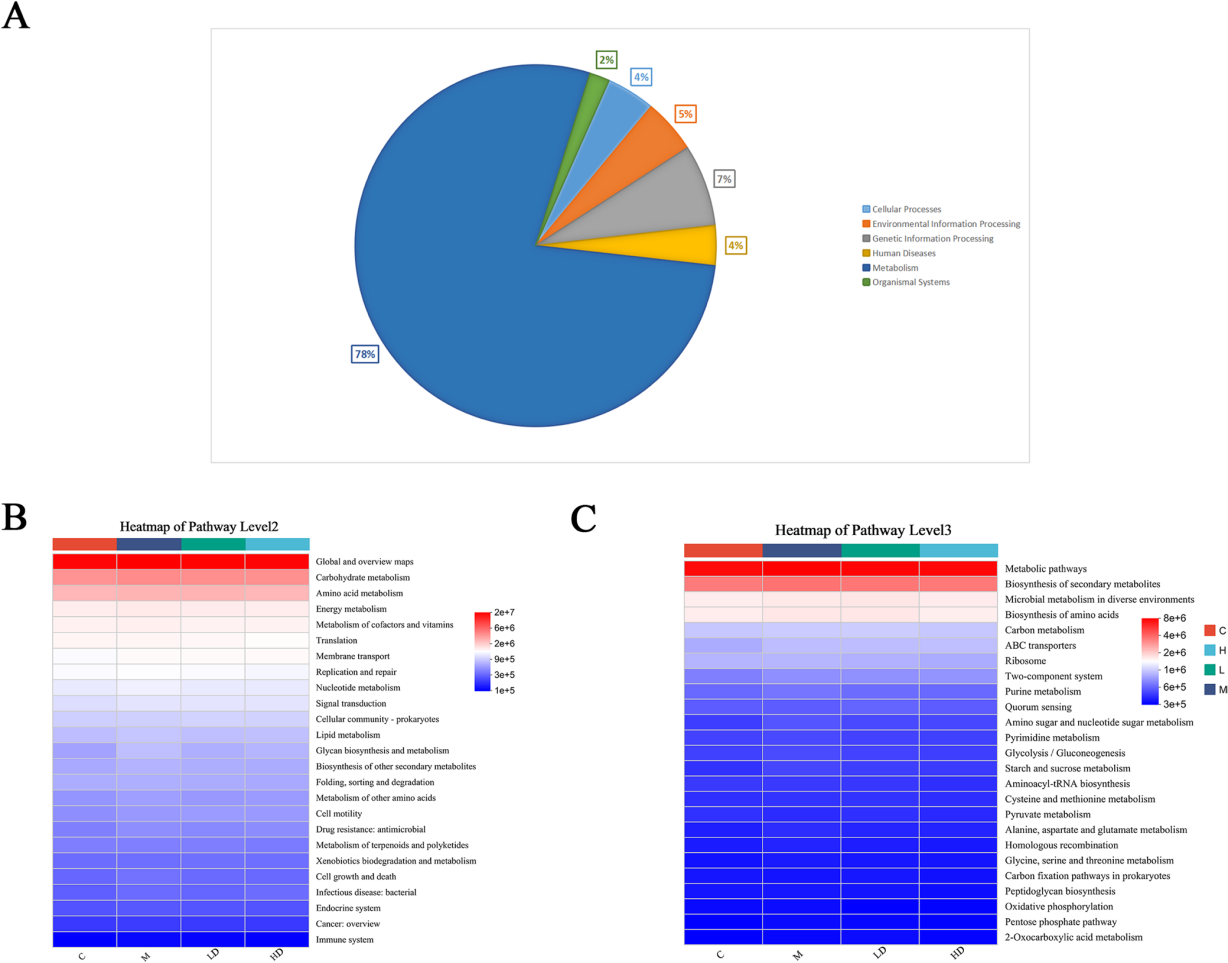
Function prediction of microbiota based on PICRUSt2 function prediction method. (A) Pie Chart of KEGG Functional Abundance Pathway Level 1. (B) Heatmap of KEGG Functional Abundance Pathway Level2. (C) Heatmap of KEGG Functional Abundance Pathway Level3.

**FIGURE 10:**
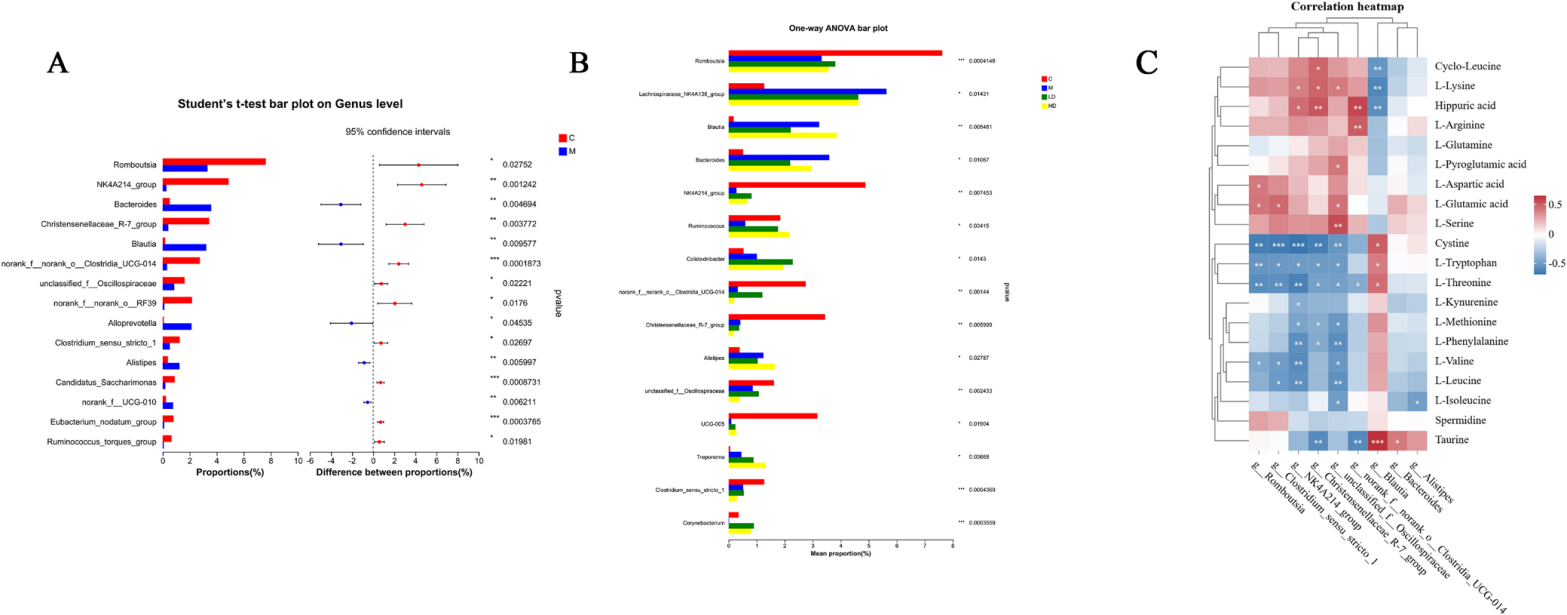
Genus level difference analysis and the relationship between amino acids and microbiota. (A) Genus with significant difference between group C and group M (B) Genus with significant difference in C, M, LD, and HD groups. (C) The relationship between amino acids and microbiota. Data are expressed as the mean ± SEM, *n* = 6. *p < 0.05, **p < 0.01, ***p < 0.001.

Metabolic pathway analysis indicated that LT might regulate amino acid metabolism to improve constipation (Figure 9). This result is consistent with the previous research of our group (Zeng et al. 2023). The amino acid content was determined by targeted metabonomics, and the method was referred to Wei et al. (2025). Further correlation analysis between the nine differential gut microbiota and targeted amino acid results from the same batch of rats (Figure 10C) revealed 14 differential gut microbiota with significant associations with amino acid results. Specifically, genera Romboutsia, Clostridium sensu stricto 1, NK4A214 group, UCG‐005, Christensenellaceae R‐7 group, and unclassified f Oscillopiraceae displayed significant negative correlations (more than four significant negative correlations). Conversely, genera Blautia and unclassified f Oscillopiraceae showed significant positive correlations (more than four significant positive correlations). These findings suggest that LT may improve constipation by modulating amino acid metabolism through the regulation of gut microbiota composition and diversity.

## 4 Discussion

Studies have shown that higher environmental temperatures and humidity, poor dietary habits, and excessive fatigue increase the likelihood of constipation during summer (Leilei and Shuran 2020; Li et al. 2023; Zhu et al. 2022). Thus, this study employed a model combining high‐fat diet with a hot and humid environment and incorporated single‐day fasting and daily running interventions to simulate modern lifestyle habits of poor diet and excessive fatigue in summer, creating a complex constipation rat model (Liu et al. 2019; Lu et al. 2024; Wang et al. 2021). After modeling, the rats exhibited typical constipation symptoms such as yellowed fur, reduced food intake, lethargy, smaller and drier feces. Compared to the control group, the model group rats showed a decreased colonic propulsion rate (*p* > 0.05) and significantly reduced fecal water content (*p* < 0.05), confirming the successful establishment of the constipation rat model.

Following LT intervention, the fecal water content of constipation rats significantly increased. This suggests that improving colonic water transport is one of the main mechanisms through which LT alleviates constipation. Aquaporins (AQP) are crucial for colonic water transport, and upregulation of AQP expression can disrupt the colonic water transport system, leading to constipation (Kon et al. 2015). KEGG enrichment analysis and the “component‐target‐pathway” network identified the PI3K/Akt signaling pathway as a significant route through which LT alleviates constipation. Additionally, research has shown that AQP3 expression levels are closely related to the activity of the PI3K/Akt signaling pathway. Wang, Lin, et al. (2024) found that AQP3 activates the H_2_O_2_/Syk/PI3K/Akt signaling pathway by regulating NOX4‐derived H_2_O_2_ transport in HeLa cells. Another study showed that red ginseng polysaccharides inhibit the PI3K/Akt pathway by downregulating AQP3, promoting ferroptosis in gastric cancer cells (Wang, Guan, et al. 2024). Unlike these findings in cervical and gastric cancer cells, in this study, LT treatment led to increased phosphorylation levels of Akt and decreased AQP3 protein expression in the colonic tissue compared to the model group. This is consistent with Zhang et al. (2023), who observed that the Nourishing Yin and Moistening Dryness Formula could activate the PI3K/Akt signaling pathway and downregulate AQP3 to alleviate Yin deficiency constipation. Notably, when a PI3K inhibitor was added to the treatment group, p‐Akt expression levels were significantly reduced, and AQP3 expression in the colon significantly increased compared to the treatment‐only group. This indicates that LT, by activating the PI3K/Akt signaling pathway, downregulates AQP3 expression in the colonic tissue, thereby improving constipation symptoms. However, the specific regulatory mechanisms of the PI3K/Akt signaling pathway and AQP3 in different tissue types or pathological states require further investigation.

Despite the observed improvement in colonic propulsion rate with LT treatment, statistical analysis did not show significant differences, suggesting that the primary mechanism of LT in alleviating constipation may not involve direct stimulation of colonic motility. Network pharmacology results suggest that inflammation may be a key target for LT's effects on constipation. Constipation is often associated with systemic inflammation (Di Rosa et al. 2023; Shatri et al. 2023), and inflammation is closely related to constipation, leading to impaired water metabolism and exacerbating the condition (Haichun et al. 2023; Yadav et al. 2009). The “constipation‐inflammation” interaction creates a vicious cycle (Sun, Zhang, et al. 2023b). In this study, both HE staining and immunohistochemistry results showed widespread inflammatory cell infiltration and lymphocyte aggregation in the gastrointestinal tissues of the model group rats, whereas LT treatment significantly reduced the levels of these inflammatory factors, indicating a notable anti‐inflammatory effect of LT.

Furthermore, gut microbiota is closely related to the occurrence and development of constipation (Yao et al. 2022). The experimental results showed that the gut microbiota structure of the model group rats was disrupted, with a significant decrease in microbiota richness. Additionally, gut microbiota imbalance can lead to chronic inflammatory responses and increased susceptibility to viral and bacterial infections. A reduced Firmicutes/Bacteroidetes ratio may increase inflammation sensitivity (Gao, He, et al. 2022). Combined with pathological observations of the colon, minor mucosal abnormalities and compromised colonic wall integrity may be related to the reduced abundance of butyrate‐producing bacteria. Butyrate plays a crucial role in maintaining colonic wall integrity, and sufficient butyrate can help prevent intestinal leakage by assisting tight junction proteins (Wang et al. 2018). At the genus level, a significant decrease in the relative abundance of butyrate‐producing genera such as *Eubacterium nodatum* group, *Ruminococcus torques* group, and Clostridium sensu stricto 1 was observed in the model group. LT intervention improved the Firmicutes/Bacteroidetes ratio, significantly increased the Sob index, and enhanced gut microbiota richness (Gao, Hu, et al. 2022), effectively reversing the dysbiosis and alleviating gut inflammation. Functional prediction results indicated that gut microbiota might regulate amino acid metabolism to improve constipation. Specific amino acids such as arginine, taurine, and methionine have been shown to promote gastrointestinal motility (Gao et al. 2023; Lee et al. 2019), while phenylalanine and glutamate contribute to improving gut barrier function (Liu et al. 2022). Additionally, amino acids such as glycine, lysine, and threonine exhibit potential therapeutic effects on constipation (Kim et al. 2019; Wang et al. 2023). Abnormal tryptophan metabolism can lead to altered 5‐HT secretion, impairing colonic circular muscle activity and suppressing motility, resulting in constipation (Blonska et al. 2024; Wang et al. 2022). Correlation analysis of differential gut microbiota and amino acids revealed significant negative correlations between g_NK4A214 group and kynurenine and tryptophan (Chojnacki et al. 2024). g_norank f_norank o_Clostridia UCG‐014 showed a significant positive correlation with arginine. In this study, Romboutsia was negatively correlated with tryptophan, but in other studies, it was positively correlated (Yebei et al. 2024). Blautia is positively correlated with taurine, and there is evidence that the diversity of intestinal microflora can be enriched by regulating taurine metabolism (Wang et al. 2025). Some researchers have found that imported chromocyte mitochondrial bioenergetics can lead to Trp‐Kyn metallic balance (Sun, Wang, et al. 2023a). However, the specific regulatory mechanism between flora and amino acids is complex and diverse, which needs further study. Moreover, g_NK4A214 group exhibited significant correlations with ten amino acids, potentially serving as a key microbial marker for LT's improvement of constipation. LT may effectively improve constipation by regulating gut microbiota metabolism and amino acid metabolism.

In this study, the correlation between the PI3K/Akt pathway and AQP3 was clarified, revealing that the g_NK4A214 group may be the key flora for LT to improve constipation. LT directly increased the water content of feces, enriched intestinal flora and improved amino acid metabolism. Compared with LT, probiotics and dietary fiber do not directly increase fecal water content in non‐pharmaceutical management. Probiotics can improve constipation mainly by inhibiting the production of harmful bacteria and affecting intestinal peristalsis (Dimidi et al. 2017), and their clinical safety needs to be studied (Daniali et al. 2020). Dietary fiber mainly improves constipation by increasing defecation times (Xu and Xue 2024), accompanied by abdominal distension. LT has great advantages in improving constipation due to its multiple mechanisms and safety.

However, due to the lack of targeted research on amino acid‐related metabolites, it is impossible to further analyze the correlation and better reveal the way of intestinal flora participating in amino acid metabolism. In addition, the animal model can't be perfectly applied to people, and whether the benefits of LT to humans are consistent with those of rats needs to be verified. In the follow‐up study, we can focus on the key effective components by establishing the correlation analysis between components and curative effect, so as to provide more basis for gradient dose selection in clinical trials. In the future, we will explore other potential signal pathways to fully understand the mechanism of LT and determine new methods to improve constipation. Through these studies, we hope to provide a more comprehensive theoretical basis and practical guidance for the treatment of constipation.

## 5 Conclusion

This study preliminarily demonstrates that LT intervention effectively alleviates symptoms of constipation in rats, with PI3K/Akt being a primary mechanism by which LT improves constipation. LT activates the PI3K/Akt signaling pathway and downregulates AQP3, which, in turn, improves gut microbiota dysbiosis and regulates amino acid metabolism to relieve constipation. These results indicate that LT holds significant potential for improving constipation and could serve as a functional food for the treatment and prevention of constipation.

## Author Contributions

**Wenxin Yu:** conceptualization (lead), data curation (lead), formal analysis (lead), writing – original draft (lead). **Jing Xue:** writing – review and editing (lead). **Zehua Yang:** writing – review and editing (supporting). **Xiaoxin Xie:** data curation (equal), formal analysis (equal). **Yi Feng:** funding acquisition (equal), writing – review and editing (equal). **Yu Zeng:** funding acquisition (supporting), writing – original draft (equal).

## Funding

This study was supported by the Guangxi Major Science and Technology Special Project (Gui AA20302018‐17) and Special fund of the State Key Laboratory of Dampness Syndrome of Chinese Medicine of the Second Affiliated Hospital of Guangzhou University of Chinese Medicine (SZ2022KF04).

## Disclosure

Author statement. All data were generated in‐house. All authors agree to be accountable for all aspects of this work.

## Conflicts of Interest

The authors declare no conflicts of interest.

## Data Availability

Data will be available upon reasonable request.
